# Proceedings of Maryland and District of Columbia Dental Association. Annual Meeting

**Published:** 1881-04

**Authors:** 


AMERICAN JOURNAL

OF

DENTAL SCIENCE.

Vol. XIV. THIRD SERIES—APRIL, 1881. No. 12.

### ARTICLE I.

### *Proceedings of the Maryland and District of Columbia Dental Association.—Annual Meeting.*—(CONTINUED.)

THIRD DAY, *Morning Session.*

The President called the Association to order at 11 o'clock. The minutes of the previous meeting were read and approved.

Dr. Noble proposed the name of Dr. R. B. Winder, Jr., of Baltimore, as a member of the Association, who was unanimously elected.

On motion of Dr. Hunt, the name of Dr. H. F. Smithe was transferred from the roll of Active to Honorary membership.

The committee to whom was referred the President's Address, made the following report:

The special Committee, to whom the President's address was referred, respectfully recommend that all that portion of it treating of the relation of Dental Societies and Associations to Dental Colleges, be referred to the Section on Dental Education and Literature for a report thereon at our next annual meeting.

The Committee further recommend that the suggestion of the President with referrence to a Mass meeting, be carried into effect, and submit the following resolutions:

*Resolved*, That a Mass meeting of the Dentists of Maryland be called to be held in Baltimore on the second day of the next annual meeting of this Association.

*Resolved*, That in the meantimet he State of Maryland be thoroughly canvassed in order to secure the co-operation of the members of the Profession of Maryland in procuring the passage of a Dental law and their attendance at this Mass meeting.

*Resolved*, That a committee of three be appointed with instructions and powers to carry into effect the foregoing resolutions.

R. Finley Hunt,

T. W. Coyle,

W. C. Thompson.

On motion the report was adopted.

Dr. Winder moved that the President of this Association be relieved from his duties as Ex-officio Chairman, being a member of this Committee. Adopted.

The Publication Committee reported as follows, which report was adopted:

The Publication Committee have to report that arrangements have been made through the kindness of Prof. Gorgas, with Messrs. Snowden and Cowman, of Baltimore, Maryland, to print the proceedings of this Association, which promise they satisfactorily performed without charge for the last meeting.

H. M. Schooley, *Ch'n ex-officio.*

J. B. Hodgkin.

Dr. Waters moved a vote of thanks be tendered these gentlemen for their kindness in printing these proceedings. So ordered.

On motion, the Secretary was requested to notify Prof. Gorgas and Messrs. Snowden and Cowman of the action taken by the Association.

The Secretary was also directed to tender a vote of thanks by letter to the Hon. Wm. A. Fisher for the kind interest he displayed in trying to get a bill passed for the better protection of Dentists through the Legislature of Maryland. Also to Mr. W. N. Smith.

Dr. Hunt made a statement about the bill that was laid before Congress for the protection of Dentists in the District of Columbia. He also made mention of the National Association that was formed in New York last summer.

Dr. Winder said he thought it necessary to remind the members of this Association that it is possible at least, that at the coming session of Congress an application may be made for an extension of the Cummings patent, and moved that the committee appointed at the last meeting to watch the proceedings of Congress and report if such attempt should be made, be continued. Adopted.

Dr. Thompson read the following paper by Dr. Laurence Turnbull, of Philadelphia:

*Hydrobromic Ether or Bromide of Ethyl as an Anæsthetic.*

The hydrobromic ether or bromide of ether was discovered by Serullas in 1827, but received no special attention until Dr. Thomas Nunnelly, of Leeds, made some experiments with it on animals in 1849. Dr. Nunnelly brought the subject again before the profession, by a paper at the meeting of the British Medical Association in 1865, in which, in conjunction with another anæsthetic, he says he had employed the one or the other in all the principal operations at the Leed's General Eye and Ear Infirmary. This was at the time when chloroform held such complete sway in England, that no importance was attached to Nunnelly's experience or experiments, and he had no one to follow him in using it, and we hear no more of it until 1876, when some experiments were made with it in France, by Rabuteau, on the lower animals, but evidently without a knowledge of the fact that this had been done previously in England by Nunnelly.

I then took the agent up without the knowledge of the experiments of Dr. Nunnelly, of England, and had it made in Philadelphia by Professor Remington, and with two friends began experimenting in September, 1877, using it first on myself, and then upon my patients. After satisfying myself as to its efficiency and safety as an anæsthetic, I laid the subject before the Pennsylvania State Medical Society in 1878, and a record of ten cases, with my conclusions, which were published in the volume of their Transactions for that year. In August, 1878, I brought it before the British Medical Association at Cork, and in September of the same year, I presented a report of one hundred cases before the International Medical Congress at Amsterdam (to which I was a delegate from the American Medical Association,) up to March, 1879, when the second addition of my work on anæsthetics went to press. I had published a report of twenty-five successful cases in quite a variety of surgical operations, and had not only employed it at my daily ear clinic, but also in the Jefferson Medical College Hospital, and administered it in April, 1879, to a patient of Dr. Samuel W. Gross, at the public clinic, when he (Dr. Gross) removed a hyoid cist in front of the neck of a child. Dr. R. J. Levis, who was at this clinic, for the first time saw it employed, and became much interested in its use.

I thus compelled chemists to make it, by producing a demand for it, and gave them, through Dr. Green, a good formula free from phosphorous; I interested surgeons all over the country to try it, and especially the surgeons of this city, by bringinfi it in every way before their attention. Subsequently the whole number of cases in which it has been employed by myself and friends up to the present time, June, 1880, will number eight or nine hundred.

I cannot but feel disappointed that two deaths, not produced by it, should have been associated with it,* as such

* The bromide of ethyl as an anæsthetic, by Marion Sims, M. D., LL. D., New York Medical Record, April 3, 1880.

accidents will be employed by those having a prejudice against the ether, to condemn it on theoretical grounds. It is my firm conviction that although in several instances recently the use of this anæsthetic has been attended with persistent vomiting, in the hundreds of cases in which it has been employed, chiefly in Philadelphia, in not one single instance has it caused cerebral trouble, or any of the symptoms produced by the action of free bromine, which are as follows. When dogs are confined in an atmosphere of bromine vapor, they suffer a profuse secretion from the eyes, nostrils and fauces, with cough, hoarseness, dyspnœa. I have experimented upon frogs, cats, dogs rabbits, and various other animals by subjecting them to an atmosphere highly charged with the vapor of hydrobromic ether, and in no instance was there the slightest irritative effects as described above.

PHILADELPHIA, June 2, 1880.

"Deputy Coroner Beam made an investigation of the circumstances, as reported in *The Times* nearly a week ago, of the death of William Linderman, eighteen years old, of Schuylkill county, while upon the operating table at the Jefferson College Hospital on Wednesday last, under the influence of the new anæsthetic, bromide of ethyl, and about to be treated for stone in the bladder. He had been about sixteen weeks under the care of Dr. R. J. Levis, one of the strongest advocates of the new anæsthetic, and was taken to the hospital by his direction. Linderman's health was very poor at the time. Dr. Ames, who applied the bromide, said no incision had yet been made, but Dr. John B. Roberts said it had. The patient was in such a condition that something had to be done, because he could not survive the hot weather; 96° 98° in the shade.

Dr. J. G. Lee, the Coroner's physician, testified that he found the brain congested, *the lungs far advanced in consumption, and the kidneys and liver enlarged* and two large encysted stones in the bladder. His opinion was that they could not have been safely taken out. *Linder-*

*man could not have lived over a week or two at any rate.* Dr. Lee said further, that he had experimented with the bromide on animals without bad results. In his opinion death resulted from exhaustion and prostration, the result of phthisis. The jury took the same view in their verdict."

In subjecting the new anæsthetic to this most severe test we do not think our friend Dr. Levis was doing justice to it; knowing the extreme debility of the patient, and that the most simple nervous shock would render him liable to death. Hundreds of patients have thus died. Again, when ordinary ether, chloroform, or other anæsthetics cause fainting, which was no doubt the result in this case, artificial respiration has to be resorted to; now we were reliably informed that when this useful means was resorted to by alternating and relaxing the chest walls, *the pus which was in this man's lungs was forced into his bronchial tubes and suffocated him.*

In some recent experiments on animals I crowded four ounces (the quantity stated to have been used by Dr. Sims) upon a dog for several minutes, by means of a tin inhaler, until he became apparently dead, with no perceptible action of the heart or lungs, but his expression of eye was clear, and the pupil was dilated, while there was no secretion from the eyes or nostrils. The apparatus was removed in the space of four minutes, and he was exposed to the air when at once he began to breathe, and at the end of the six minutes, he had almost entirely recovered consciousness.

The dog did not seem much inclined to move for ten or twelve minutes afterwards. While this dog was only partly under the influence of this anæsthetic, having at first caught the inhaling apparatus with his under teeth; there was a great deal of rigidity, and slight tetanic movements of the extremities but this was overcome by the free use of the ether. Now, had we been using chloroform, just before we would have been ready to perform any experiments upon the animal, he would have been dead, and no removal of the anæsthetic nor the introduction of atmospheric air, would have been of any avail. Again if Squibb's rectified, and absolute ether had been employed; we must have super-saturated the animal, and been annoyed by the expectoration of large quantities of mucus. Then we frequently have seen tetanic convulsions, requiring several assistants to hold the patient, with great reduction of temperature, from the use of ordinary ether. The rapidity of the anæsthetic action of hydrobromic ether and its rapid elimination from the system by the lungs, are two of its chief merits for all operations that are not prolonged. If an operation is to be very tedious, and requires from one to two hours, we would advise the additional use of purified sulphuric ether to the anæsthetic. *We would therefore recommend pure hydrobromic ether in operations not lasting over forty minutes.* There is one great advantage in the use of this agent, that the administrator must attend to the anæsthetic all the time, he cannot watch the operation and forget the patient for a few seconds; his whole attention must be given to keep up its action. We have often felt sure that the wet napkin, from the water, in the ordinary ether pressed over the patients mouth by the weight of the body of the persons giving the ether, and watching the operation, were the indirect causes of the death of the patient. Within the last few days we have employed it in labor for the second time; and it has peculiar advantages in that it is so rapid in its effects, and the patient is comforted between the pains, but never passes into such a state of profound anæsthesia, that she is aroused by the expulsive effort, and has all her consciousness about her, and none of the depressing efforts of ether or chloroform. It is also most valuable in these cases in changing the position of the child, also in bringing forward the neck of the uterus into its proper position.. In neither of these instances was there disturbance of the bowels, pain in the back or head. To the country practitioner who has to extract teeth or perform all the minor operations in surgery it is a great boon, as it acts like nitrous oxide gas; it is well where a number of teeth are to be extracted, that a prop of hard wood attached to a string should be used, so as to prevent such an accident as once occurred in Philadelphia under the use of nitrous oxide gas, as of the swallowing of a prop of cork. In many cases where you do not want a very profound narcotism with hydrobromic ether, the muscles of the patient become rigidly contracted. This condition occurred in a recent occasion, when we administered (℥ i) of this anæsthetic and the operator's finger was caught and pinched, as also his forceps, and yet before operating we could touch the cornea with impunity. Although the impression passed away very rapidly, we extracted twelve teeth with entire success, the patient promptly recovering consciousness, and not feeling the pain. In the following case the patient went under it very kindly. This patient was a man of very nervous temperament. With three drachms of the hydrobromic ether anæsthetic was produced without any struggling, and in four minutes from the time he had commenced to inhale it, the dentist had extracted ten teeth, and he had fully recovered consciousness, although he had just eaten a heavy breakfast of solid food. There was no neusea in either of those cases.

In a recent case of cataract extraction the patient went beautifully under the influence of the anæsthetic, extraction was accomplished, and the patient recovered so as to be able to count fingers, yet owing to some strong coffee which he drank; from dyspeptic symptoms or the swallowing of water soon after the operation, she became very sick at her stomach, and vomited for nearly twenty-four hours, and yet the case did well. In a case of operation for torticolis in a woman, she swallowed so much air with the ether, that as a consequence she complained of pain, of a hysterical character, in lower part of the abdomen, the same which is often the result of nitrous oxide gas inhaled, and too much air admitted.

A few days ago we received a letter from Dr. J. Patterson Cassells, of Glasgow, a distinguished Aurist and a Surgeon to a celebrated Glasgow Infirmary ; he writes : that he has used a specimen of the hydrobromic ether, which I gave him at Cork, as vapor, in diseases of the middle ear, and have also employed it as an anæsthetic with success.

Discussion being in order, Dr. Winder said : Mr. President, I wish to say that I have seen this agent administered in quite a number of cases. It acts very rapidly and its effects pass off as rapidly. When first given to us it was claimed to produce no nausea. That is a mistake. It has been used by one or two of the first surgeons of Baltimore, and I have heard from one of them that he is not much pleased with it from actual experiment. I have used it myself in three or four cases. It produces sufficient anæsthesia, but acts rapidly and passes off as rapidly. I could not pronounce it free from danger. All these agents are dangerous. Normal respiration is absolutely necessary to continue life. In producing anæsthesia you have brought on asphyxia. I think there is no anæsthetic known that will support life. Many of them are very dangerous and the experience of numbers of our profession, added to the revelations of the spectroscope, pronounce nitrous oxide gas the most so of all. While I use them there is no man more wary or more afraid of anæsthetics. I do not consider any of them safe, nor I do not think it necessary to absolve this new anæsthetic from the category of others known to be unsafe.

Dr. Atkinson.—The responsibility of having opinions at all that are to be pronounced for other men's minds to take cognizance of, renders it impossible for me to keep still when this subject is up for discussion. All you who understand my views on anæsthetics in general and in particular know that I am opposed to them " in toto" ; and that the only advisable anæsthetic to be exercised upon a human being except in the rarest cases is that of a kind heart and clear head guiding the hand in performing operations upon the body. I have never thought them safe in hospital or private practice. Many cases have I seen where they were recorded in the Colton Dental Association as successes where the persons had been persuaded to sign their names to that certificate before they had complete use of their senses. I am so much in earnest about this thing that if it was in my power I would wipe them from the face of the earth.

Dr. Webb.—I certainly coincide with Dr. Atkinson in all he has said in regard to anæsthetics. I want to say that I stand before you to-day with weak lungs, and these lungs are weak from the use of nitrous oxide gas. Now then I would say that I earnestly believe that it would have been better for dentists and for the people if artificial teeth had never been devised. For so many would not have been prompted to rip out the teeth—these priceless pearls,—and put in an inartistic row of tombstones. Men who extract teeth knowing they should be saved, do something for which they should be put in prison; in many cases should be hung for such things.

Dr. Atkinson.—Opium is an anæsthetic that is claimed to be a boon. I think it one of the greatest curses that ever came from the east or the west. I have had patients that had acquired a habit of taking it just from having it prescribed at some time for the relief of some pain.

Dr. Winder.—The subject we are discussing is anæsthesia, not narcotics. I will agree with Dr. Atkinson and Dr. Webb in all that they have said here regarding the improper removal of teeth. It is simply outrageous malpractice. We must not arraign the anæsthetic in associations with this butchery and put the abuse upon it. It is the *improper use* of an agent that should condemn it, not the *use* for which it was made. You may take whiskey and it is the *abuse*, not the *proper use*, that condemns it. To condemn it entirely except in such abuse, would be to my mind radically wrong. I think one man's whole attention should be given to the administration of the anæsthetic and have some one else to do the extracting or other operating. I think anæsthetics are given to us by the Creator, to use, and I think it wrong in the gentlemen to condemn them in toto.

Dr. Webb.—In some major operations anæsthetics may be used with good results. If there be a chance between the patient dying under the operation and living if an anæsthetic be administered then give it, but in no minor surgical operation should it be given. I said that so far as the dentist is concerned it would have been better if the anæsthetic had not been discovered. It does lead patients to this ripping out of the natural teeth, operations they would not have had performed without these agents.

The subject of anæsthesia was passed.

The Executive Committee made the following report, which was adopted:

WASHINGTON, OCT. 29, 1880.

The Executive Committee respectfully report that they have discharged the duties imposed upon them by the Constitution, to the best of their ability.

Invitations to attend the present meeting were sent to many prominent members of the profession outside the bounds of the association, some of whom we have had the pleasure of welcoming among us.

The admirable and instructive clinics given by Drs. M. H. Webb, of Lancaster, Pennsylvania, R. R. Underwood, of West Chester, Pennsylvania, J. L. Baker, of Coatsville, Pennsylvania, and George F. Reese, of Brooklyn, New York, have been most highly appreciated by the members of the Association generally, and have added great additional interest to our meetings. The thanks of the Association are due to these gentlemen for the sacrifice of time and money they made in order to be with us and lend their valuable aid in carrying out the objects of our Association.

There have been no dental appliances presented for examination and report by your Committee.

In regard to special matter of reporting a time for the annual meeting of the Association, which was referred to the Executive Committee, they have to report: that after a full consideration of the subject, they are forced to the belief that there is no particular season of the year which could be selected for our meetings, that would prove convenient for *all* the members: but so far as the committee are able to judge, a majority of them would find it less inconvenient to leave their offices, for a few days, immediately after the usual summer holiday—say about the first week in September. The committee would therefore recommend that the first Wednesday in September, 1881, be selected as the time for holding the next meeting of the Association.

The treasurer's report, which has been examined and found correct—shows that there was a balance of $4.40 in his hands at the commencement of the present year. Received since for dues and assessments $78.00

Making a total of $82.40
now in the hands of the Treasurer.

The expenses of the present session of the Association which have been examined and approved by the Committee amount in the aggregate to $47.35
In addition to this amount there is due by the Association a balance fee for printing the proceedings of 1878, amounting to $43.80
And also the bills for expenses in the effort to obtain Legislation in regard to the practice of Dentistry in the State of Maryland, which were presented by Professor Winder, amounting to $22.18

Which makes a total indebtedness of $113.33
It will be seen that this leaves a balance of indebtedness of $30.93 in excess of the amount now in the hands of the Treasurer, which it will be necessary for the Association to provide for.

All of which is respectfully submitted.

R. B. Donaldson,
J. L. Wolf,
*Committee.*

Dr. Donaldson suggested that some arrangement to pay the expenses of the meeting be made.

The members present contributed to make up the necessary sum to relieve the Association of debt.

The rules were suspended and Drs. R. P. Fletcher and Hall Lewis, of Washington, were unanimously elected members.

On motion, the secretary was authorized to subscribe to the "American Journal of Dental Science" in the name of the society.

The society then proceeded to elect officers for the ensuing year, and Drs. Hunt and Winder were appointed tellers to conduct the same.

The election resulted as follows:

President, Dr. H. B. Noble, of Washington.
Vice President, Dr. T. S. Waters, of Baltimore.
Cor. Secretary, Dr. H. M. Schooley, of Washington.
Rep. " Dr. J. C. Smithe, of Washington.
Treasurer, Dr. J. B. Ten Eyck, of Washington.

Drs. R. B. Winder, T. W. Coyle and H. C. Thompson were appointed as Executive Committee.

The retiring President upon leaving the chair, said:

" *Gentlemen.*—I cannot leave you without an expression of opinion as to your uniform kindness and courtesy toward me in my official capacity; you have certainly made my duties by your kind assistance, sit as lightly as is possible, and thanks very cordially.

To those gentlemen who have favored us with their presence we are greatly indebted, and I but express the individual and collective opinion of the Association when I thank them very heartily for the papers they have presented; and for their clinical operations. This Association is not sectional, it reaches to all parts of our country, and all parts of the world for truths; and whoever presents an article for consideration presents it to unbiased minds, who receive it on its relative merits alone. There is, to borrow an expression from our esteemed friend Dr. Atkinson, (and I wish I could borrow a car load of condensed articulated sayings of his) "a togetherness in this Association that is apparent to all, the apartness," if any, is scarcely discernible. I now ask of you the same kindness to my successors as you have shown to me."

Drs. Hunt and Evans were appointed to conduct the newly elected President to the chair. Upon being introduced Dr. Noble said:

"*Gentlemen.*—I appreciate this honor, and feel I trust duly thankful for the honor of being elected as your presiding officer for the ensuing year. This is no time or occasion for making an address, only to thank you and ask you to overlook any failures I may make, promising to do the best in my power for the interest of the Association."

Dr. Waters upon being introduced said: "I thank you for this honor and promise to support our President to the best of my ability."

Drs. Smith and Ten Eyck each thanked the Association and promised to do the best in their power for it.

Dr. Winder moved a vote of thanks for Dr. T. W. Coyle the retiring Treasurer. Dr. Coyle thanked the Association for the compliment.

A vote of thanks was extended Dr. John Lanahan for clerical services.

The President appointed on the publication committee Prof. Gorgas, Drs. Hunt and Hodgkin. Dr. Schooley chairman *ex-officio.*

On motion of Dr. Winder a vote of thanks was extended the proprietor of the hotel, and the mover of the resolution was on motion of Dr. Hunt directed to convey the information.

On motion of Dr. Donaldson the Association adjourned *sine die.*

